# Sixth Meeting of the Association of Medical Superintendents of American Institutions for the Insane

**Published:** 1851-06

**Authors:** 


### SIXTH MEETING OF THE ASSOCIATION OF MEDICAL SUPERINTENDENTS OF AMERICAN INSTITUTIONS FOR THE INSANE.

This body met in Philadelphia, in the hall of the American Philosophical Society, on Monday, May 19th. It first met in Philadelphia in 1844, and is composed of the medical superintendents of the various institutions for the insane on this continent, and of those who have been so, and have attended meetings of the Association while in office. It has since held meetings in Washington, New York, Utica, Boston, and now again in Philadelphia.

It holds annual meetings, and examines critically any institutions for the insane, near its place of meeting, if invited by the proper authorities.

Its objects are to improve and advance the treatment of the insane; secure more extensive provision for all classes and a better style of buildings, and internal arrangements; and to make all the medical officers familiar with the results and modes of treatment of our best institutions.

The interest in the Association is on the increase, and it has already effected much good, with promise of future usefulness.

Thirty of the institutions in the U. S. and British Provinces are now connected with the Association, and it seems probable that, in a little time, no well conducted institution will be satisfied without having a representative at its meetings.

The following members were present,—Dr. Ray, of Butler Hospital, R. I.; Dr. Chandler, of Mass. State Hospital; Dr. Cutter, of Pepperill Institution, Mass.; Dr. Butler, of Hartford Retreat; Dr. Benedict, of N. York State Hospital; Dr. Earle, late of Bloomingdale Asylum, N. Y.; Dr. Nichols, of Bloomingdale Asylum, N. Y.; Dr. Ranney, of Blackwell's Island Hospital, N. Y.; Dr. Buttolph, of N. J. State Hospital; Dr. Kirkbride, of the Pa. Hospital for the Insane; Dr. Worthington, of Friends' Asylum, Pa.; Dr. Haines, of Philadelphia Lunatic Hospital, Blockley; Dr. Curwen, of Pa. State Lunatic Hospital; Dr. Fonerden, of Maryland Hospital; Dr. Parker, of South Carolina State Hospital; Dr. Hanbury Smith, of Ohio State Hospital; Dr. Patterson, of Indiana State Hospital; Dr. Higgins, of Illinois State Hospital; Dr. Jarvis, of Dorchester private institution; Dr. Smith, of Missouri State Hospital; Dr. Stokes, of Mount Hope Hospital, Ind.; and Dr. Morrin, of Quebec (Canada) Lunatic Asylum. Representatives from the Boards of Managers of several Institutions for the Insane were also present.

Invitations were received from the officers of the Mint, Blind Asylum, Deaf and Dumb, and Friends' Asylums, Pennsylvania Hospital, Pennsylvania Hospital for the Insane, Athenæum, &c.

In the absence of the President (Dr. Wm. M. Awl), Dr. Parker, of S. C., was appointed temporary chairman.

The permanent officers elected were as follows:—Dr. Luther V. Bell, of Mass., President; Dr. Isaac Ray, of R. I., Vice-President; Dr. Thos.

S. Kirkbride, of Pa., Secretary and Treasurer. Owing to the absence of Dr. Bell, Dr. Ray presided.

Dr. Awl having resigned his place, complimentary resolutions were passed, expressive of regret, and a high appreciation of his long and valued services among the insane.

A number of papers of importance were read during the session, which elicited much discussion; several of these will be published. Among them was one by Dr. Galt, on treating insane patients in buildings where there are those afflicted with other diseases. One on the use of stramonium in the treatment of insanity, by Dr. Cutter. One by Dr. Curwen, containing a manual for the use of attendants in Hospitals for the Insane. A valuable and important paper, on the course to be pursued by medical witnesses when called into Court, was read by Dr. Ray, and ordered to be published, at the special request of the Association, as containing the sentiments of the Association on the subject.

Dr. Earle read an account of many European institutions, not generally known in this country, and visited by him two years ago.

Dr. Kirkbride, from the Standing Committee on the construction of Hospitals for the Insane, read a report, embracing 27 propositions, on the subject, which were fully endorsed by the Association, and will be published in our next number, as the sentiments of the body. Dr. Morrin read a paper on Typho Mania; Dr. Chandler one on restricting Hospitals to receiving patients of one sex only. Dr. Nichols presented a paper, prepared by Dr. Williams, of N. Y., on Typho Mania; Dr. Jarvis one on the supposed increase of insanity; Dr. Kirkbride one on the best arrangements for kitchen, laundry, bakehouse, &c., in hospitals for the insane; Dr. Haines one on the warming and ventilating of Blockley Hospital.

Dr. Chandler read, agreeably to appointment, an obituary notice of the late Samuel B. Woodward, M. D., first President of the Association, and Dr. Nichols, one of Amariah Brigham, M. D., one of its Vice Presidents. These were highly interesting and will be published in the proceedings of the Association.

During the stay of the Association in Philadelphia, it was most courteously treated, and visited and examined critically the Pennsylvania Hospital for the Insane, under Dr. Kirkbride; Friends' Asylum, under Drs. Worthington and Evans; and Blockley Hospital, under Dr. Haines. The sentiments of the Association in reference to them are recorded in the following resolutions unanimously adopted, viz:

*Resolved,* That the members of this Association have visited and inspected the Pennsylvania Hospital for the Insane, under the care of Dr. Kirkbride, as well as the parent institution in the city of Philadelphia, with great interest and satisfaction, recognizing in both abundant evidence of the well directed benevolence to which they owe their origin, and feeling convinced that, if not unequalled, they are at least unexcelled.

*Resolved,* That upon a close inspection of the Friends' Asylum for the Insane, near Frankford, under the care of Drs. Evans and Worthington, the Association has much pleasure in testifying to the excellent condition in which they found that well conducted and now venerable institution.

*Resolved*, That the visit of the members of the Association to the Blockley Hospital and Lunatic Asylum, under the medical care of Dr. Haines has afforded them an opportunity of avowing their conviction that this establishment occupies a prominent position among the great charities which are the glory of Philadelphia. The cleanliness and comfort of its spacious apartments, the classification, order and freedom from restraint of its insane inmates, are commendable, and in all that relates to the supply of water, warmth, ventilation and drainage, this institution is not only in advance of similar pauper establishments, but even of some of our State Hospitals.

*Resolved*, That while the Association finds so mnch to admire and commend in this institution, and approvingly observes the astonishing improvements effected since its last meeting in this city, six years ago, it feels free to remind the Board of Guardians of its well known opinions on the importance of providing labor and spacious and constantly and readily accessible grounds for exercise for the Insane; especially as the institution possesses abundant means of accomplishing such advisable improvements in the extensive grounds and beautiful gardens connected with it.

*Resolved*, That our thanks are especially due to the Board of Managers and Guardians of all the Institutions above mentioned for their personal attentions and kindness shown us, on the occasion of the visit of inspection to which reference has just been made, as well as at other times, and for other proffered privileges of which want of time has prevented our availing ourselves.

*Resolved*, Tuat our thanks are due and are hereby tendered to the President and Directors of the Girard College for Orphans, for the liberal manner in which the Association was entertained during its visit to every part of that magnificent and admirably conducted institution.

*Resolved*, That the Association returns its warmest thanks to Dr. R M. Patterson, Director, and Franklin Peale, Esq., Chief Coiner of the U. S. Mint, for the highly appreciated privilege afforded us of inspecting every part of this establishment, justly renowned for the elegance and perfection of its machinery and arrangements, and for the admirable manner in which it is conducted.

*Resolved*, That the Association gratefully acknowledges the liberality and kindness which prompted the American Philosophical Society to tender the use of its hall for the meetings of the Association, and the use of which places us under special obligations to that body.

*Resolved*, That the thanks of the Association are also due to the Managers of the Pennsylvania Hospital for the offer of their beautiful Library Room for the meetings of the Association; to the officers of the Academy of Natural Sciences, for the privilege of making a very gratifying visit to that valuable institution; to the officers of the Pennsylvania Institution for the Instruction of the Blind; of the Pennsylvania Institution for the Deaf and Dumb; of the Athenæum, and of the University of Pennsylvania, for their courteous invitations to visit those institutions, which want of time alone prevented our accepting.

*Resolved,* That the Secretary be requested to furnish the daily papers of Philadelphia, with a copy of these resolutions for publication.

They also in a body visited several other public institutions.

Various important resolutions were adopted—among them were, one in reference to the proper provision for the insane poor; one on the effects of early education in producing insanity; and one relating to Miss Dix and her land bill.

Dr. Kirkbride resigned the post of Secretary to which Dr. Buttolph was appointed.

The committee for reporting the time and place of the next meeting, reported in favor of New York city, on the 3d Tuesday of May, 1852, which was affirmed by the Association.

Dr. Nichols, of New York, submitted a series of resolutions, tendering the acknowledgments of the Association to the different scientific and humane institutions of Philadelphia for their kindness and courtesy.

A resolution was adopted tendering thanks to Dr. Ray for the manner in which he had presided and discharged his duties.

Another resolution was passed, regretting the resignation of Dr. Kirkbride, and complimenting him upon the faithful performance of his duty.

A committee, consisting of Dr. Bell, chairman, and Drs. Haines and Smith, were appointed to report at the next meeting upon the subject of heating and ventilating.

Dr. Benedict offered a resolution to publish the proceedings of the Association in the Journal of Insanity and other journals.

Dr. Ray read a few remarks in reference to the reception of the Association in this city.

Dr. Bell made some remarks in relation to the organization of the Association in 1844; the doubts then entertained of its ability to sustain itself; and its increasing and now triumphant success.

At 12 o'clock M., the Association adjourned, *sine die,* May 23.

